# In Vitro Study of the Interaction of Innate Immune Cells with Liquid Silicone Rubber Coated with Zwitterionic Methyl Methacrylate and Thermoplastic Polyurethanes

**DOI:** 10.3390/ma14205972

**Published:** 2021-10-11

**Authors:** Franziska Woitschach, Marlen Kloss, Karsten Schlodder, Alexander Borck, Niels Grabow, Emil C. Reisinger, Martina Sombetzki

**Affiliations:** 1 Department of Tropical Medicine and Infectious Diseases, Center of Internal Medicine II, University Medical Center Rostock, 18057 Rostock, Germany; franziska.woitschach@uni-rostock.de (F.W.); marlen.kloss2@uni-rostock.de (M.K.); emil.reisinger@uni-rostock.de (E.C.R.); 2 Biotronik SE & CO. KG, 12359 Berlin, Germany; karsten.schlodder@biotronik.com (K.S.); alexander.borck@biotronik.com (A.B.); 3 Institute for Biomedical Engineering, University Medical Center Rostock, 18119 Rostock, Germany; niels.grabow@uni-rostock.de

**Keywords:** monocytes, inflammation, implant material, macrophage polarization, foreign body reaction, biocompatibility, zwitterionic methyl methacrylate

## Abstract

The biocompatibility of medical devices, such as implants and prostheses, is strongly determined by the host’s immune response to the implanted material. Monocytes and macrophages are main actors of the so-called foreign body reaction. The innate immune system macrophages (M) can be broadly classified into the pro-inflammatory M1-type and the anti-inflammatory, pro-healing M2-type. While a transient inflammatory initial state can be helpful during an infection, persistent inflammation interferes with proper healing and subsequent regeneration. The functional orientation of the immune response, mirrored by monocyte polarization, during interaction with different biomaterials has not yet been sufficiently explored. In implant manufacturing, thermoplastic polyurethane (TPU) represents the state-of-the-art material. The constantly growing areas of application and the associated necessary adaptations make the optimization of these materials indispensable. In the present study, modified liquid silicone rubber (LSR) were compared with two of the most commonly used TPUs, in terms of monocyte adhesion and M1/M2 polarization in vitro. Human monocytes isolated from venous blood were evaluated for their ability to adhere to various biomaterials, their gene expression profile, and their cytokine release. Based on the results, the different polymers exhibit different potential to bias monocytes with respect to early pro-inflammatory cytokine production and gene transcription. Furthermore, none of our test materials showed a clear trend towards M1 or M2 polarization. However, we were able to evaluate the inflammatory potential of the materials, with the classic TPUs appearing to be the most unreactive compared to the silicone-based materials.

## 1. Introduction

Implant materials used in the cardiovascular system consist of modified biological substances, metals, and polymers. The implantation of synthetic and natural polymers is becoming increasingly important in modern medical care. Nowadays, polymer materials have a very high value in cardiac surgery. Owing to their good biocompatibility and adaptable mechanical properties (e.g., high ductility, good resistance to abrasion or chemicals, high flexibility), they can be used as prostheses, e.g., for artificial vessels, heart valves, and heart support systems [1].

Cardiovascular implant materials are not only in contact with blood but also in close contact with tissue. Therefore, foreign body reactions between the material surface and surrounding stressed tissue and recruited immune cells can decisively determine the success or failure of an implant [2]. The foreign body reaction is divided into two phases: (1) an inflammatory phase immediately after implantation, in which pathogens are neutralized and damaged tissue is removed; and (2) a reparative phase, which involves healing and restoration of the implant site by regeneration of the damaged tissue [2]. Monocytes are instrumental in this host inflammatory and foreign body reaction to biomaterials [3,4]. Immediately after implantation, the surface of the material comes into contact with blood, which further promotes cellular adhesion and activation. An inflammatory response is initiated and monocytes migrate to the tissue–material interface. Once attached to the surface of the implant, they mature into a macrophage phenotype and can further fuse into multinucleated foreign body giant cells. Both phases, the inflammatory and wound healing phase, are orchestrated by a variety of cell types, such as neutrophils, fibroblasts, endothelial cells, and platelets. However, neutrophils are the predominant cell type at the implantation site in the acute inflammatory stage which are replaced by monocytes within the early stage, and, in the chronic inflammation stage, it is the monocyte-derived macrophage (MDM) [3,5]. MDMs are an integral part of the initial inflammatory response and the general response to the implanted biomaterial, and they determine whether a fibrous capsule forms or the inflammatory process subsides and thus whether tissue regeneration occurs [6]. 

The cytokines produced by these cells in the early stage of the foreign body reaction affect the recruitment and activation of further leukocytes. A deeper understanding of how biomaterials control inflammatory response is important for the development of implants that integrate well with host tissue to promote healing and restore functionality to the affected tissue. This helps to inhibit the formation of fibrous tissue, which improves the integration and performance of the implant to fulfil its intended function [7]. It can be assumed that an initial inflammatory reaction can never be completely prevented after the insertion of an implant. However, it must come to an end soon. Therefore, it is of importance that biomaterials are less attractive for monocyte attachment, attenuate the pro-inflammatory response, and promote polarization into M2-type macrophages.

Silicones are widely used as biomaterials for medical devices, such as liners in prosthetics, orthopedic implants, breast implants, and extracorporeal equipment. For certain applications, other materials are too weak or too cost-intensive [8]. Silicone is used due to their excellent thermal and biocompatibility properties. It has low viscosity, controllable crosslinking, and easy implementation. Therefore, it is used as scaffold material in many cases. With its ease of fabrication, nontoxicity, high flexibility, and transparency, it is a very suitable material for developing highly customizable implants [9]. 

To prevent monocyte attachment, polymers with anti-adhesive properties are preferred for the manufacture of implants. Due to these properties, poly-methylmethacrylate (PMMA) is widely used in medical procedures and has been investigated in various studies. It is known as a suitable biodegradable candidate for medical implants, e.g., as a material for intraocular lenses and as a bone filler or in the manufacture of dental prostheses [10,11,12]. Further research on the optimization of PMMA with, for example, chitosan or silver nanoparticles has been conducted [11,12,13]. The wide range of applications underlines the high biocompatibility and, thus, the useful application of PMMA in diverse medical fields. Optimization of biomaterials with the aim of preventing implant-associated infections is an essential part of research. Surface modification by zwitterions represents a promising approach aimed at anti-adhesive properties. Modifications containing zwitterions reduce the initial attachment of non-specific proteins to the material, which allows subsequent attachment of, for example, bacteria [14]. One of the best characterized zwitterions is 2-methacryloyloxylethyl phosphorylcholine (MPC). MPC is a methacrylate with a phosphorylcholine group in the side chain which forms a zwitterionic structure of a phosphate anion and a trimethylammonium cation. Zwitterions, such as MPC, have great potential due to their anti-fouling properties, which are generated by an interplay of two mechanisms. The first effect is the formation of a hydration shell based on electrostatic interactions, which hinders the binding of proteins to the surface. Secondly, steric hindrance is achieved by the zwitterionic polymer chains due to their hydrophilicity and mobility [15,16]. The advantages of non-fouling zwitterionic materials, such as PMMA-MPC, include their simple synthesis, ease of application, abundance of raw materials, and availability of functional groups [16]. Kojima and colleagues have shown that the amount of adsorbed natural phospholipids was increased on MPC copolymers [17]. Ishihara et al. on the other hand investigated that MPC side chain with a poly(n-butyl methacrylate) backbone which suppresses the adhesion, aggregation, as well as activation of blood cells after contact with human citrated whole blood [18]. In another study, they investigated that modification of polysulfone with MPC reduced protein adsorption and platelet adhesion [19]. We were able to show another positive effect. In our previous work, we could show that PMMA-MPC has a significant anti-biofilm formation effect on *S. aureus* [20].

Another class of polymers with anti-adhesive properties are the thermoplastic polysulfones. Polysulfonate (PSU) and poly(1,4-phenylene-ether-ether-sulfones) (PPSP) are widely used in production of membranes for ultrafiltration [21]. The hydrophobic properties, i.e., high pH value as well as temperature resistance, also qualify these materials for implant manufacturing. Moreover, the additional ether compounds of PPSP cause improved mechanical and chemical properties [22].

Despite their wide application as biomaterials, PMMA-MPC, PSU, and PPSP have not yet been used for cardiovascular implants, but are worth closer investigation due to their suitable properties. The aim of this study was to investigate (1) monocyte activation and differentiation into macrophages; and (2) the influence of biomaterial surface chemistry on cytokine production and gene expression of monocyte/macrophage population. 

## 2. Materials and Methods

### 2.1. Polymer Functionalization

**Figure 1:**
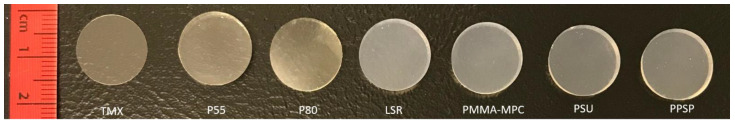
Photographs of all materials used in this study.

Two different implant materials, a thermoplastic polyurethane (TPU) and a platinum-cured liquid silicone rubber (LSR), were subjected to comparative analyses. TPU samples were prepared by extruding a film from the TPU pellets using a twin-screw extruder with a flat film nozzle. Round samples with a diameter of 16 mm were cut out. Two TPUs, TPU 55 and TPU 80, were used, which differ in material strength, with 55 having a higher hardness than 80. To process the LSR base material, the polymer was filled in a flat mold and vulcanized with a hot press. After a post-curing process in the oven, round samples with a diameter of 16 mm were cut out. The samples were coated differently. PMMA-MPC coating: LSR samples were swollen and treated with methyl methacrylate (MMA), methacrylic acid, azoisobutyronitril (AIBN), and methacryloyloxyethyl phosphorylcholine (MPC). The samples were then rinsed with deionized water. PSU coating: LSR samples were treated with PSU-DMAC (dimethylacetamid) solution. PPSP coating: LSR samples were treated with PPSP-DMAC solution. After coating, all samples were cured in a drying oven, washed in a surfactant solution. and underwent a sterilization process with ethylene oxide (ETO). Handling of the samples was performed in an aseptic environment. The samples were analyzed for absence of soluble endotoxins with the Limulus amoebocyte lysate (LAL) test. Endotoxin levels for all materials were beneath 0.2 EU/mL. For further specifications of all investigated materials, see Woitschach et al. [20]. Discoidal samples of thermanox (TMX), a well-characterized polyester, were used as reference material. TMX is widely used in cell attachment experiments, due to its hydrophilic surface. Therefore, we used it as a positive reference material. Macroscopic images of all materials are shown in Figure 1. The following abbreviations are used throughout the manuscript: thermanox = TMX, TPU 55 = P55, TPU 80 = P80, unmodified LSR = LSR, LSR + PMMA − MPC = PMMA − MPC, LSR + PSU = PSU, and LSR + PPSP = PPSP.

### 2.2. Endotoxin Measurement

To exclude endotoxin contamination of our materials, the PierceTM Chromogenic Endotoxin Quant Kit (ThermoFisher, Dreieich, Germany) was used. One specimen of each material was placed in a 24-well culture plate and incubated overnight in sterile water with endotoxin levels of <1 EU/mL. The water from each material was then used in triplicates and analyzed for measurement according to the manufacturer’s instructions.

### 2.3. Human Monocyte Isolation and Cultivation

Buffy coats were obtained from healthy adult donors from the German Red Cross (Berlin, Germany), and peripheral blood mononuclear cells were isolated by density gradient centrifugation using Histopaque (1.077 g mL^−1^, Sigma-Aldrich, Darmstadt, Germany) as previously described [21,22]. In brief, buffy coats were diluted 1:1 with 1× Earle’s Balanced Salt Solution (EBSS), layered over gradient medium (Histopaque), and centrifuged unrestrained at 400× *g* for 30 min at room temperature. The turbid layer, containing lymphocytes and monocytes, on top of the density gradient, was collected into fresh 50-mL tubes. To wash the obtained cell suspension, three volumes of 1×EBSS buffer was added, mixed gently by inverting, and centrifuged at 300× *g* for 10 min at room temperature. Each cell pellet was resuspended in 1 mL of medium, and cells were counted using Casy cell counter (CASY TT, OLS Omni Life Science, Bremen, Germany).

All cells were cultured in RPMI 1640 without phenol red (ThermoFisher, Dreieich, Germany) supplemented with 1% penicillin/streptomycin and 10% fetal bovine serum (FBS) (ThermoFisher, Dreieich, Germany). For viability testing, 5 × 10^5^ were used. For cytokine and gene expression analyses, 1 × 10^6^ were analyzed. To prevent cell attachment aside from the plates, cells were suspended in 100 µL of media and seeded in 24-well culture plates (Nunc multidish, ThermoFisher, Dreieich, Germany) directly onto the different 16-mm-diameter material disks. Half an hour later, an additional milliliter medium was added. After 2 h, the cells were washed by careful aspiration and fresh medium was added. The remaining heterogeneous cell population enriched in monocytes was cultured for 12 h, 3 or 6 d at 37 °C and 5% CO_2_. In addition, the same amount of cells was seeded in 24-well culture plates with LPS (100 ng/mL) or IL-4 (20 ng/mL) as controls for M1 and M2 polarization, respectively. The medium was replaced by fresh medium every three days. Each experiment was performed six times with independent primary donor material (n = 6), and 2 technical replicates for each measurement.

### 2.4. Adhesion and Morphology of Monocytes/Macrophages

The isolated human monocytes were cultured on the different biomaterials for 12 h (h, short-term) and for 3 and 6 days (d, long-term). To determine the degree of adherence of the monocytes/macrophages, the supernatants of the cell cultures was taken 12 h after incubation and an automatic count was performed with the Casy cell counter. 

### 2.5. Viability and Metabolic Activity

After 12 h, monocyte viability was determined by calcein AM staining (Thermo Fischer, Waltham, MA, USA). Intracellular esterases of living cells convert the non-fluorescent calcein AM into green fluorescent calcein after hydrolysis of the acetoxymethyl ester. The cell supernatants were removed to eliminate the unattached cells. Next, 40 µL of a 2 mg/mL calcein stock solution was diluted in Hanks’ Balanced Salt Solution (HBSS) and gently mixed. One ml of the staining solution was added to the cells and incubated at 37 °C for 30 min. The fluorescence was visualised using a fluorescence microscope (Axio Scope.A1, Zeiss, Germany) equipped with a camera (AxioCam MRc, Zeiss, Jena, Germany).

After 12 h, the monocytes were treated with 10% CellQuanti-Blue^TM^ reagent and incubated at 37 °C for 5 h. The reagent contains the non-fluorescent redox dye resazurin, which is converted into the highly fluorescent product resorufin when reduced by metabolically active cells. To determine the metabolic activity, the optical density of the cell suspensions was measured hourly using a 530-nm excitation filter and a 590-nm emission filter in a microplate reader (FLUOstar Omega, BMG Labtech, Ortenberg, Germany). For viability, the endpoint measurement after 5 h was used. The materials itself cannot degrade resazurin, as evidenced by the lack of metabolic activity in the cell-free controls.

### 2.6. Cytokine Quantification

To determine cytokine production, monocytes were cultured for 12 h, 3 d, and 6 d on the different material disks. Cytokines in cell-free supernatants were quantified using enzyme-linked immunosorbent assay (DuoSet ELISA Kit, R&D Systems, Minneapolis, MN, USA) for IL-4, IL-6, IL-10, IL-12, CCL2, TNF-α, and TGF-β according to the manufacturer’s instructions.

### 2.7. Gene Expression Quantification

Isolation and purification of mRNA was performed using RNA-Isolation Mini Kit (Qiagen, Venlo, The Netherlands). The mRNA was quantified by spectrophotometric measurement (Colibri, Berthold Technologies, Bad Wildbad, Germany). A total of 500 ng of total RNA was reverse transcribed using a High-Capacity cDNA Reverse Transcription Kit (Thermo Fisher, Waltham, MA, USA), according to the manufacturer’s instructions. Assessment of gene expression levels was performed using two-step quantitative reverse transcription PCR. For RT-qPCR, 50 ng of cDNA was amplified in a total reaction volume of 10 μL, using TaqMan Universal MasterMix II and primers (TaqMan Gene Expression Assay, Thermo Fisher, Waltham, MA, USA) for the following genes: interleukin-6 (*IL6*, Hs00174131_m1), interleukin 1b (*IL1b*, Hs01555410_m1), tumor necrosis factor-α (*TNFα*, Hs00174128_m1), monocyte chemotactic protein 1 (*MCP/CCL2*, Hs00234140_m1), CD206 (*MRC1*, Hs00267207_m1), interleukin-10 (*IL10*, Hs00961622_m1), transforming Growth Factor β (*TGFβ1*, Hs00998133_m1), acyl-malonyl condensing enzyme 1 (*AMAC1/CCL18* Hs00268113_m1), suppressor of cytokine signaling 3 (*SOCS3*, Hs01000485_g1), transglutaminase 2 (*TGM2,* Hs01096681_m1), and vascular endothelial growth factor a (*VEGFa*, Hs00900054_m1). Glyceraldehyde 3-phosphate dehydrogenase (*GAPDH*, Hs99999905_m1) was used as an endogenous control. Relative fold change in gene expression was obtained using the 2^−ΔΔct^ method. The data were presented as the fold-change to TMX normalized to the reference gene expression level of GAPDH.

### 2.8. Statistics

Statistical analysis was performed using GraphPad Prism 5.0 (GraphPad Software, La Jolla, CA, USA). Values are expressed as mean + SE_mean_. Normal distribution was tested using the D’Agostino and Pearson Omnibus Normality Test. Non-normally distributed samples were compared using the Kruskal–Wallis test followed by a Dunn’s post hoc test. For all statistical analyses, *p* values < 0.05 were considered significant. * *p* < 0.05, ** *p* < 0.01, *** *p* < 0.001.

## 3. Results

### 3.1. Decreased Monocyte Adhesion on Thermoplastic Polyurethane (TPU) and Liquid Silicone Rubber (LSR) with Only Minor Effects on Cell Viability and Metabolic Activity

**Figure 2:**
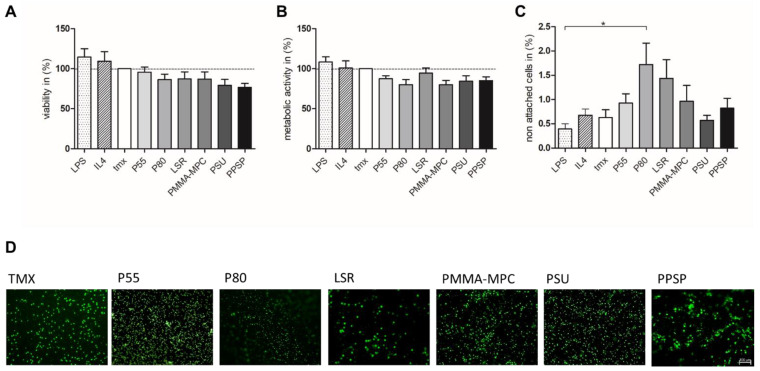
Decreased monocyte adhesion on thermoplastic polyurethane (TPU) and liquid silicone rubber (LSR) with only minor effects on cell viability and metabolic activity. Then, 5 × 10^5^ cells were incubated on the materials (**A**) Viability was measured after 12 h by treating the cells with 10% CellQuanti-BlueTM reagent and incubated for 5 h at 37 °C. (**B**) Metabolic activity was calculated by measuring the optical density of the cell suspensions every hour. (**C**) Non-attached cells were determined after 12 h of incubation by collecting the supernatants and counting the non-attached cells with Casy cell counter. (**D**) 1 × 10^6^ cells were incubated on the different materials and analyzed after 12 h by microscopy by calcein AM. Magnification, ×2400. Data are represented as mean + SE_Mean_ of six independent experiments. Significant results are indicated as * *p* < 0.05.

Twelve hours after seeding fresh human monocytes, the proportion of non-surface adherent cells in the culture supernatant was determined. The range of non-surface attached cells was 0.5–2% (5000–20,000 cells/mL). The lowest number of cells was recovered from the supernatants of TMX and PSU. The comparatively higher cell numbers in the supernatants of P80 and LSR indicate lower monocyte/macrophage adherence (Figure 2C). This is consistent with the calcein AM staining (Figure 2D). As shown by stronger fluorescence signal on the TMX, P55, PMMA-MPC, PSU, and PPSP materials, there are more cells on the materials here, indicating a lower number of unattached cells. On the different materials, no reduction in cell viability could be detected compared to TMX (Figure 2A). The average metabolic activity of the seeded cells on the materials was reduced by 10–20%, with the exception of LSR, but without significant differences compared to TMX. LSR showed only a slight decrease (Figure 2B). 

### 3.2. Unmodified and Modified Liquid Silicon Rubbers (LSR) Trigger an Unpolarized Immune Response

**Figure 3:**
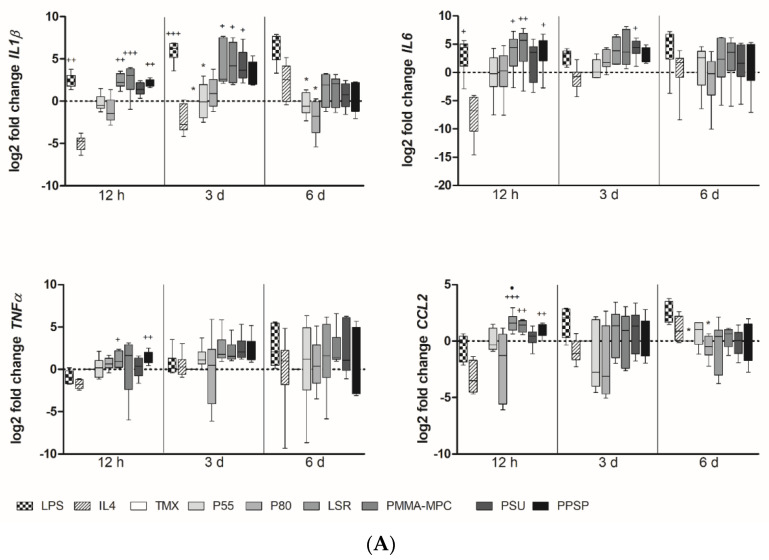

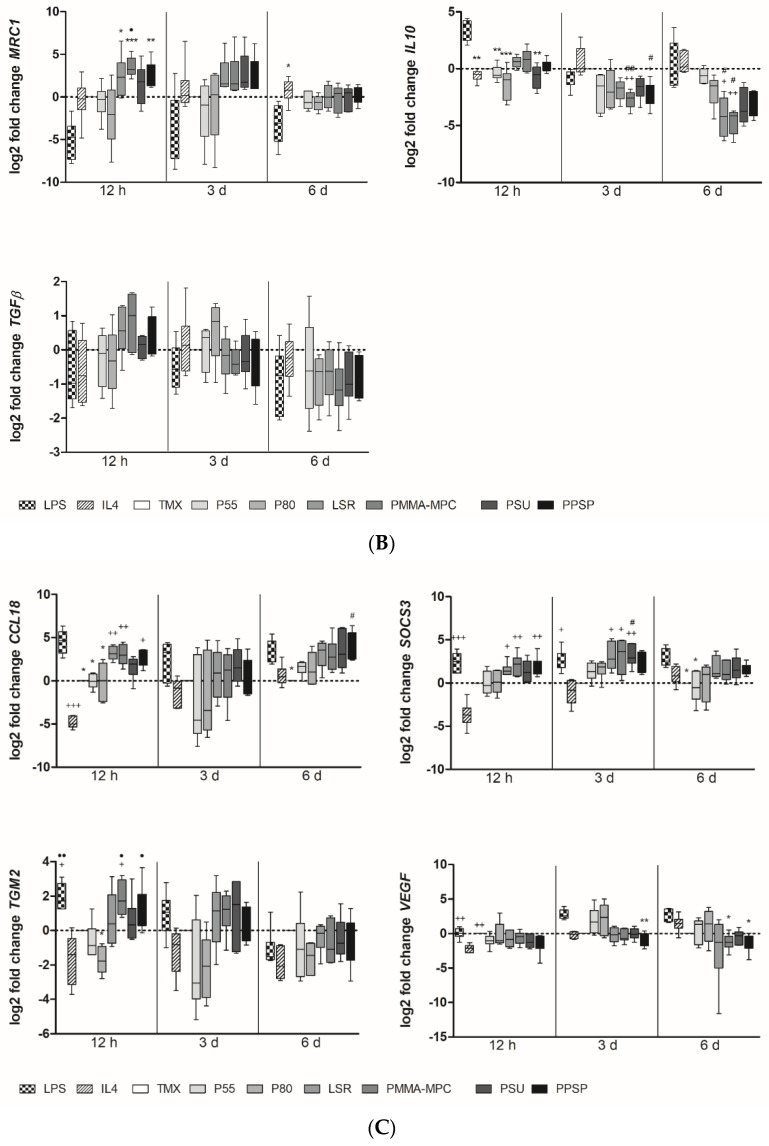
Unmodified and modified liquid silicon rubbers (LSR) trigger an unpolarized immune response. (**A**) Relative gene expression of *IL1b*, *IL6*, *TNFα* and *CCL2* was determined by real-time PCR. (**B**) Relative gene expression of *MRC1*, *IL10* and *TGFβ* was determined by real-time PCR. (**C**) Relative gene expression of *CCL18*, *SOCS3*, *TGM2* and *VEGF* was determined by real-time PCR. Data are represented as mean + SE_Mean_ of 6 independent experiments. Significant results are indicated as * *p* < 0.05; ** *p* < 0.01; *** *p* < 0.001 vs. LPS; + *p* < 0.05; ++ *p* < 0.01; +++ *p* < 0.001 vs. IL4; # *p* < 0.05; ## *p* < 0.01 vs. TMX; • *p* < 0.05; •• *p* < 0.01 vs. P80.

For M1 phenotyping, we analyzed expression levels of *IL1b, IL6, CCL2, TNFα*, and *IL12*. We found a tendency towards higher expression levels for *IL-1b* and *IL-6* in monocytes/macrophages on silicone-based materials compared to TPUs (Figure 3A). *IL-1b* expression peaked after 3 d, whereas *IL-6* expression came up earlier at 12 h. The *TNF-α* expression of monocyte/macrophages peaked after 6 d of incubation on all materials to be tested, with exception of P80. Both TPUs show a rather low potential to induce the expression of M1-associated genes in monocytes compared to the silicone-based materials. Only the late expression of *TNF-α* by monocytes/macrophages on P55 and the late expression of *CCL2* on P55 are remarkable (Figure 3A). For M2 phenotyping, we selected the genes *IL-10, TGF-β,* and *MRC1*. PMMA-MPC induced significantly higher expression of *MRC1* compared to both TPUs (Figure 3B). Although not significant, *IL-10* and *TGF-β* levels were enhanced in macrophages on LSR and PMMA-MPC after 12 h (Figure 3B). *CCL18* expression was significantly upregulated in our analysis on LSR samples in comparison to TPUs (Figure 3C). *SOCS3* expression in macrophages was increased on LSR, PMMA-MPC, and PSU after 3 d, in contrast to TPUs (Figure 3C). *TGM2* expression was increased on LSR, PMMA-MPC, PSU, and PPSP, but not on P55 and P80 (Figure 3C). We observed high expression for *VEGF* after 3 d for both TPUs and no increase in expression on the LSR materials (Figure 3C). 

### 3.3. Unmodified and Modified Liquid Silicone Rubber (LSR) Stimulates Macrophages to Ssecrete Proinflammatory Cytokines

**Figure 4:**
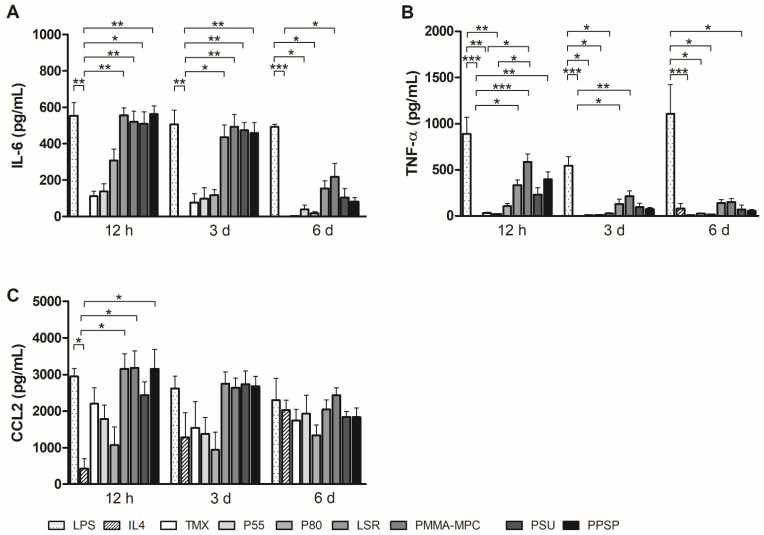
Unmodified and modified liquid silicone rubber (LSR) stimulate macrophages to secrete pro-inflammatory cytokines. Cytokine production of (**A**) IL6 (**B**) TNFα (**C**) CCL2 was measured by using enzyme-linked immunosorbent assay. Data are represented as mean + SE_*Mean*_ of six independent experiments. Significant results are indicated as * *p* < 0.05; ** *p* < 0.01; and *** *p* < 0.001.

**Figure 5:**
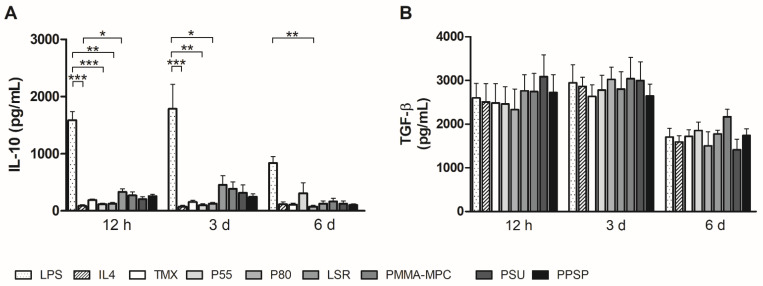
Unmodified and modified liquid silicon rubber (LSR) induce anti-inflammatory cytokine production. Cytokine production of (**A**) IL10 (**B**) TGFβ was measured by using enzyme-linked immunosorbent assay. Data are represented as mean + SE_Mean_ of six independent experiments. Significant results are indicated as * *p* < 0.05; ** *p* < 0.01; and *** *p* < 0.001.

To further characterize their ability to control and mediate the immune response, we also measured the cytokine production of human monocytes/macrophages on the different biomaterials. The IL-6 production by monocytes/macrophages on all silicone-based materials was significantly enhanced compared to IL-4. In particular, at the two earlier time points (12 h and 3 d), there was a strong release of IL-6 on these materials. Lower levels of IL-6 were produced by monocytes and macrophages on P55 and P80 (Figure 4A). A similar picture emerged for TNF-α. However, the high release is limited to the 12 h time point. However, the silicone-based materials induce a higher TNF-a release than the two TPUs (Figure 4B). Monocyte chemotactic protein 1 (MCP1/CCL2) was also most highly expressed at the 12-h time point (Figure 4C). Monocyte/macrophage on LSR materials was higher than in the LPS-stimulated sample. Secretion of regulatory IL-10 was increased in the supernatants of all silicone biomaterials (LSR, PMMA-MPC, PSU, and PPSP) at earlier time points (12 h and 3 d) compared to the reference TMX. Monocytes/macrophages on the TPUs secreted comparable amounts of IL-10 as on TMX, with the exception of increased IL-10 levels on P55 after 6 d. All values were above those of the IL-4 sample as a positive control (Figure 5A). Increased TGF-β secretion could be measured on all materials at the two earlier time points (Figure 5B).

## 4. Discussion

In the phase of acute inflammation, which usually subsides within a week, activated M1 macrophages are characterized by the expression of the cytokines IL-12, IL-6, TNF-α, IL-1b, and CCLs [23,24,25]. The increased expression of M1-associated cytokines, such as IL-6 and TNF-α, by monocytes/macrophages on the silicone-based materials shown here is an important indication that the innate immune system responds to these materials, as both are well-known mediators of acute phase inflammation [26,27]. This effect is most pronounced after 12 h and 3 days, indicating a high initial potential to stimulate pro-inflammatory immune responses (Figure 4A,B). This is consistent with the findings of Bhaskar et al., who have shown that silicone triggers pro-inflammatory immune response [28]. Regarding PMMA-MPC, our results contrast with those of Quin et al. In their study, they observed that PMMA-MPC soft gels have lower cell adhesion, exhibit a lower potential to trigger pro-inflammatory macrophage activation, and promote M2-like polarization [29].

Four to seven days after implantation, monocytes infiltrate and differentiate into macrophages with different polarization. This plays a crucial role in immune regulation and the wound healing process. The typical cytokine pattern of M2 macrophages consists of IL-10, mannose receptor C-type 1 CD206/MRC1, and TGF-β [23,24,30]. IL-10 is capable of suppressing cytokine expression by a variety of cell types, e.g., macrophages, NK cells, and T lymphocytes [25,31]. Therefore, the higher release of IL-10 by monocytes associated with LSR might indicate suppression of the immune response at early time points ranging from 12 h to 3 days (Figure 5A). Another M2 marker, MRC1, responds in the same pattern, suggesting a stronger initial stimulation of the M2 phenotype, which, however, does not become established until 6 days (Figure 3B). CCL18 is produced by monocytes and plays an important role, in part, by activating the immune system and by inducing tolerance and homeostasis under steady-state conditions. In addition, CCL18 promotes the maturation of macrophages to phenotype M2, which in turn promotes immunosuppression and healing [30,32]. Higher expression levels of CCL18 were observed in the LSR samples at the last time point we determined (Figure 3C). The comparatively low gene regulation and cytokine expression in IL4-stimulated cells may be due to a lower binding affinity of monocytes for IL4 compared with that of monocyte-derived macrophages [26]. 

The expression of the SOCS3 gene paralleled the expression of pro-inflammatory genes (Figure 3C). Suppressors of cytokine signaling (SOCS) are proteins that affect immune response by cytokine receptor binding and thus can regulate signaling pathways [27]. The translational importance of increased SOCS3 levels in inflammation is illustrated by evidence of their involvement in human M1 polarization [30]. Immune cells release pro-fibrogenic factors, such as platelet-derived growth factor (PDGF), VEGF, and TGF. This leads to fibrotic tissue and encapsulation of the implant, which impairs implant function. This process makes up the foreign body reaction. VEGF expression is greatly increased after 3 days in the TPUs compared to the unmodified and modified LSRs (Figure 3C). Wound-healing cytokines, such as VEGF, perform a reparative role by promoting neovascularization in the tissue surrounding the implant. It is expressed by M1 and M2 macrophages, but treatment with VEGF induces upregulation of the M2 marker and downregulation of the M1 marker [31]. The decrease in VEGF expression after 6 days in monocytes/macrophages on TPUs and the absence of VEGF on LSRs argues against a regenerative effect. Rapid and adequate vascularization is crucial for the long-term success of implants. Native vascular endothelium provides a non-thrombogenic surface and prevents intimal over-proliferation. A confluent endothelial cell layer on material surfaces is widely considered an approach to improve the biocompatibility of implanted cardiovascular materials. Therefore, VEGF coatings or functionalization have been used to protect stents from in-stent restenosis, induce angiogenesis, or protect small diameter vascular grafts from thrombosis or degeneration by inducing endothelial formation [32,33,34]. However, VEGF can also lead to increased neo-intimal hyperplasia [34], and there are implants where integration with the surrounding tissue should be avoided, such as transvenous pace maker electrodes. 

## 5. Conclusions

In the present study, two different commonly used TPUs (P55, P80), unmodified LSR, and three modified LSRs (PMMA-MPC, PSU, PPSP) were evaluated for their potential to modify the immune response by activating and polarizing human monocytes (M1 or M2). The different polymers were shown to have different potential to affect monocytes in terms of early pro-inflammatory cytokine production and gene transcription. Furthermore, none of the new candidates material tested showed a clear trend towards M1 or M2 polarization. However, we were able to assess the inflammatory potential of the materials, with the classic TPUs appearing to be the least sensitive compared to the silicone-based materials.

Whether the in vitro results shown are actually associated with a stronger post-implantation reaction must subsequently be clarified in vivo. What is clear, however, is that the materials tested have a different potential to activate monocytes/macrophages (inflammation, wound healing, endothelial activation), which is of great importance with regard to the intended use.

## Data Availability

The datasets generated for this study are available on request to the corresponding author.
